# Supplementary material for: Supplementing sleep actigraphy with button pressing while awake
Source: PLoS One. 2020 Jun 18;15(6):e0234060. doi: 10.1371/journal.pone.0234060 (PMC7302569; doi:10.1371/journal.pone.0234060)
Supplement: S2 File — Comprehensive technical information on our self-developed vibrating wristband. (DOCX) [file pone.0234060.s002.docx]

# Vibration Wristband Setup

## Technical Details on the self-developed vibrating wristband device used in the study “Supplementing sleep actigraphy with button pressing while awake”.

A compact programmable low energy micro “Arduino Gemma” device, Adafruit Industries, NY was employed.

Technical specifications according to the manufacturers’ website: https://www.arduino.cc/en/Main/ArduinoGemma

| Microcontroller | ATtiny85 |
| --- | --- |
| Operating Voltage | 3.3V |
| Input Voltage | 4V-16V |
| Digital I/O Pins | 3 |
| PWM Channels | 2 |
| Analog Input Channels | 1 |
| DC Current per I/O Pin | 20 mA |
| Absorption | 9 mA while running |
| Flash Memory | 8 kB (ATtiny85) of which 2.75 kB used by bootloader |
| SRAM | 512 Bytes (ATtiny85) |
| EEPROM | 512 Bytes (ATtiny85) |
| Clock Speed | 8 MHz |
| Diameter | 27.94 mm |

Also, the following parts were used:

- 1 NPN Transistor Type PN2222
- 1 Diode Type 1N4001
- 1 Resistor 620 Ω
- 1 Coin battery holder for 2x CR 2032 coin battery and ON/OFF switch (output voltage 6V)
- 1 Mini Disc vibration motor (for full Datasheet see: https://cdn-shop.adafruit.com/product-files/1201/P1012_datasheet.pdf )
  Properties:
- Model: 10B27.3018; Number: 100614
- Diameter 10mm, Thickness 3mm
- Rated Voltage: DC 3.0V
- Rate Speed at Rated Voltage and Rated Load: 11000±3000rpm
- Rated Current 75mA or less
- Weight: 1,2g
- Mechanical Noise: 50dB(A) Max at 3.0V
- Vibration amplitude: 1.0G (at 3.0V)
- Voltage used: 0.8V
- Resulting frequency and approximate amplitude: 80Hz at about 0.3G

The parts were configured as shown in the following circuit diagram:


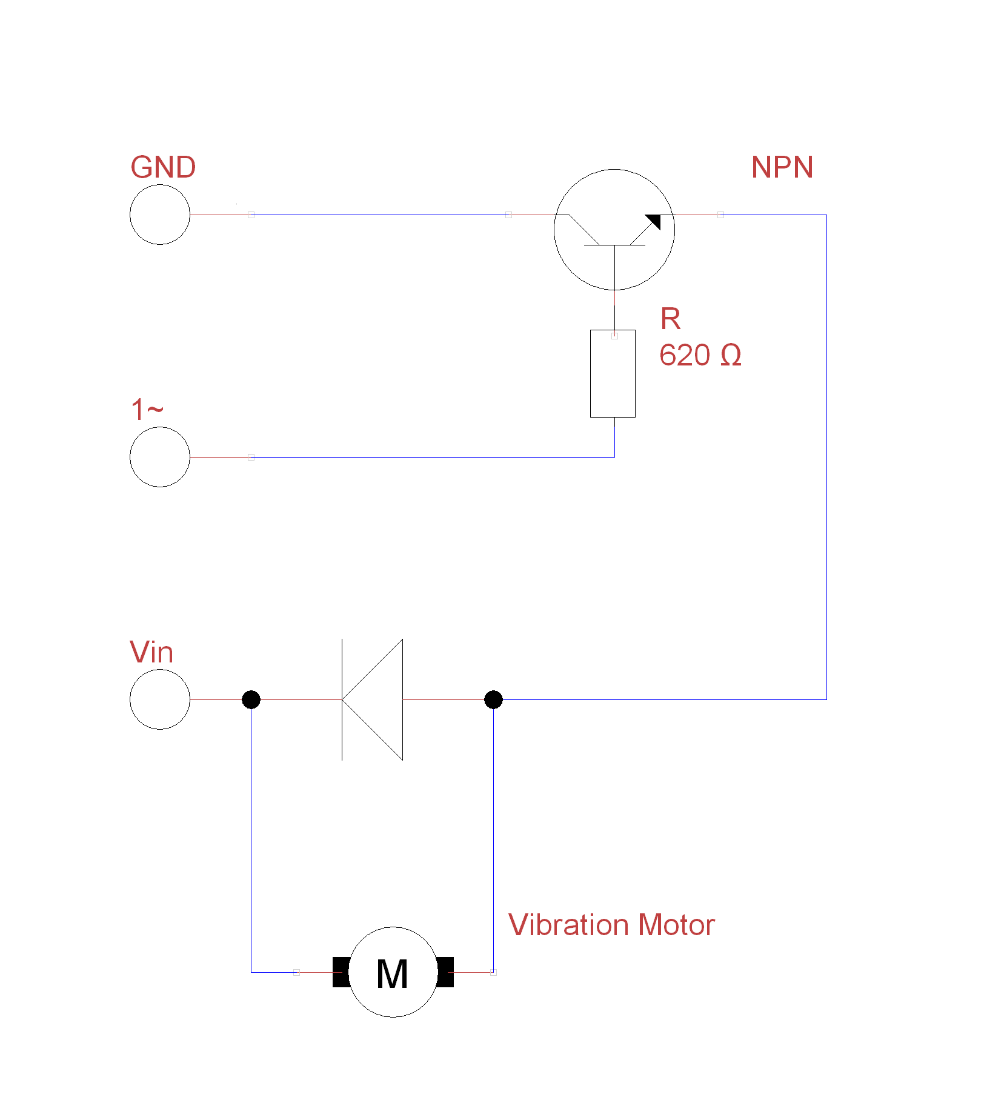


The three pins connect to the microcontroller board. The vibration motor was glued to its back. To protect the micro controller mechanically, it was enclosed in a rubber case.

This system and the battery holder are sewn a regular sweat wrist band used in sports, the vibration motor facing the inner side.

### Code:

The following program code was loaded onto the device:

const uint32_t onTime = 2 * 1000L; // Vibration motor run time, in milliseconds

const uint32_t interval = 5 * 60 * 1000L; // Time between reminders, in milliseconds

const uint32_t intensity = 38; // Vibration intensity value from 1 to 255

const uint32_t offTime = interval - onTime;

void setup() {

pinMode(1, OUTPUT);

pinMode(0, INPUT_PULLUP);

pinMode(2, INPUT_PULLUP);

DIDR0 = _BV(AIN1D) | _BV(AIN0D); // Digital input disable on analog pins

analogWrite(1, intensity); //startup-sequence signaling “on”

delay(100);

analogWrite(1, 0);

delay(100);

analogWrite(1, intensity);

delay(100);

analogWrite(1, 0);

delay(100);

analogWrite(1, intensity);

delay(100);

analogWrite(1, 0);

delay(100);

analogWrite(1, intensity);

delay(100);

analogWrite(1, 0);

delay(100);

analogWrite(1, intensity);

}

void loop() {

pinMode(1, OUTPUT);

analogWrite(1, intensity);

delay(onTime);

analogWrite(1,0);

pinMode(1, INPUT);

delay(offTime);
