# Supplementary material for: Supplementing sleep actigraphy with button pressing while awake
Source: PLoS One. 2020 Jun 18;15(6):e0234060. doi: 10.1371/journal.pone.0234060 (PMC7302569; doi:10.1371/journal.pone.0234060)
Supplement: Attachment — Submitted filename: Response to Reviewers.docx [file pone.0234060.s003.docx]

**Response to Reviewers**

**“Supplementing sleep actigraphy with button pressing while awake”** Ref.: PONE-D-20-03712

Journal Requirements:

1. Please ensure that your manuscript meets PLOS ONE's style requirements, including those for file naming.

**We checked the manuscript for compliance with PLOS One’s style requirements and applied changes on the title page and where else necessary. Table 2 was reformatted to meet the guidelines for tables.**

2. Thank you for stating the following in the Acknowledgments Section of your manuscript:
"This research was supported by the Department of Veterans Affairs Sierra-Pacific Mental Illness Research, Education, and Clinical Center (MIRECC)."

We note that you have provided funding information that is not currently declared in your Funding Statement. However, funding information should not appear in the Acknowledgments section or other areas of your manuscript. We will only publish funding information present in the Funding Statement section of the online submission form.

Please remove any funding-related text from the manuscript and let us know how you would like to update your Funding Statement. Currently, your Funding Statement reads as follows:
"The authors received no specific funding for this work."

**The research was not funded by a grant. The funding statement remains applicable. We clarified the modalities of the support we received by MIRECC. The Acknowledgement section now reads as follows:
*“The Department of Veterans Affairs Sierra-Pacific Mental Illness Research, Education, and Clinical Center (MIRECC) provided office space and technical supplies.*“ (L352-353)**

Reviewers' comments to the Autor:

Reviewer #1:
The study aims to investigate the impact, on sleep parameters obtained by actigraphy, of pressing an event-button when lying without any movement, but not in a drowsy state. Two system have been compared. In the first one, the button had to be pressed every 5 min when an haptic signal (vibrations throughout a wristband) is given. In the second one, instead of being reminded to press the button by a vibration, lying awake was to be signalled by pressing the marker about once every 5-10 min.

Results show that the first condition was unacceptable for about one out of two study participants while in the second one, is acceptable to many. The uncertainty on estimation of sleep onset while lying-awake is reduced compared to actigraphy without pressing a button. In epoch-by-epoch analysis, both technics detect more (3.4-8.2%) wakefulness than in actigraphy alone, with about two time more in the wristband stimulation.

This is an interesting paper, which have some relevance for interpreting the results obtained by actigraphy in sleep studies. However, sleep might be slightly affected by the instruction and this disturbance might be dependant of the type of patient recorded. One of the limit of the study is to have include any adult regardless of health status. The next step will be to compare the results obtained in young good sleepers (in which actigraphy have a very good correlation with polysomnography) and other population (olders, insomiacs, …).

**We intentionally included a diverse set of participants with no limitations in terms of health status in order to increase generalizability of our findings. However future investigations should address particular populations.**

Nonetheless, self-initiated button pressing to indicate being awake while lying in bed is acceptable to many, interferes little with sleep on average, and adds to the information given by actigraphy, this will certainly change the way this instrument is used.

*Reviewer #2:*
As the authors point out, it has been known that autographs, while very useful in describing several sleep parameters, seriously misrepresent SOL and wakefulness during TIB. Supplementary, well validated behavioural measures could improve the accuracy of describing sleep and wakefulness during the night. The authors have chosen to use spontaneous and cued button presses for this purpose. While the paper provides a useful addition to the current literature, a number of suggestions follow which might clarify several points and perhaps extend the usefulness of the paper.

The introduction is well written and the goals of the study are clearly related to the experimental design. (An additional reference is needed on L 71.)

***We included the missing reference [11] (L69).***

Sound ethics and consent procedures were employed. The use of diverse participants increases the generalizability of the findings. Behavioural definitions are clearly described and the MotionLogger is a good choice for an actigraph. A programmable vibratory wrist band is a good way to deliver a cue for a behavioural response, but the physical parameters (Hz, amplitude) must be included for replicability. In such studies, the faintest cue possible should be used.

**We specified the physical parameters more clearly (L93-95). Also, we added a supplementary file (S2 File) comprising all technical information on the self-constructed wristband.**

Please state clearly that this study was conducted at home. L. 99 "usually consecutive" nights is imprecise. Elaborate.

**We now state that the recordings were made at home (L98). We added the missing information on “usually consecutive” nights in the “Procedure” section (L102-105).**

In the Results, important information is omitted. The reader ought not to need to calculate overall compliance (131/180 nights = 73%: 31/60 for RP = 52%; 51/60 for SP = 85%; 49/60 for NP = 82%).

**We added the missing information in the “Results” section (L188-189). Twenty-nine subjects were included in the study, which we clarified under “Participants” (L76).**

The authors address the issue of the acceptability of the two button pressing conditions and found no significant differences between conditions, which is very fortunate, for they should have used a counter-balanced design in which there was an equal probability of any participant receiving any condition on any given night. In general, the results are thoughtfully analyzed and well presented.

**We did find lower acceptance rates for RP (L196, L200 and L221). However, our headings were somewhat misleading and we modified them. We also added a Fisher’s Exact Test for overall compliance under RP (L199-200).**

**We are aware of the disadvantageous design of giving the three conditions in a predefined order rather than randomizing the conditions over the course of the six nights. We deliberately decided to do so in order to keep the protocol simple and understandable for the subjects.**

Under Actigraphic Measures (L 230-234) the description of differences in SOL is not clearly presented. It is important to fully consider why SOL might be longer in the cued response condition (RP) than in the non-cued conditions. Authors need to go back to the definitions of their three conditions to fully explain their data.

**Actigraphically measured SOL is longer in both conditions involving button presses, the cued and the non-cued, compared to NP. We made this clearer by rewriting this section (L235-239).
We now comment on the necessity of future experiments involving PSG to ascertain whether or not this reflects a real delay in SOL or is solely explained by artifactual movements (L239-240). Actigraphically measured sleep variables were not different between RP and SP. We added this statement in L240-241.
We expanded Table 2 by adding a column comparing actigraphic measures under RP vs. SP to give a more comprehensive view, and rearranged and reformatted Table 2 to enhance readability and understandability. Statistical tests and significant results are now highlighted.**

Marker measures: L235-43 Possible explanations - or that the marker measures index different points on the W/S/W continuum.

**Thank you for this suggestion. We now address this issue (L251-255).**

This comment also applies to the next section on Simultaneous actigraphic vs marker measures. Also, in L 246, it is stated that RP underestimated SOL, but data from Table 2 shows the opposite - which is more in line with expectations. Please check the remainder of Table 2 against interpretations. Also, the section is written as if the autograph is the Gold Standard for measuring sleep. I thought that the author's premise was that additional behavioural measures might fine-tune sleep measurement. Please be consistent with your own Introduction. Please re-write indicating which measures give the shortest to longest estimates of SOL.

**We clarified the ambiguous wording and now clearly describe the results in line with Table 2 (L256-259). Also, we consistently corrected the wording here (L256-259) and in the discussion, (L329-332) to not give the impression that actigraphy is our gold standard.**

If you want to compare against a Gold Standard, repeat the study including polysomnography, focussing on EEG changes and look to see what percentage of the variance between EEG and behavioural measures can be accounted for with various weightings of behavioural measures. This also applies to interpretation of other measures.
L 285 - The fact that the SP condition shows only moderate agreement suggests that this measure may acc our for additional variance when describing W and S and therefore may indeed be a useful metric.
The Discussion is well written and argued.
Further to L.315, normally, lying still is a prerequisite for falling asleep and remaining so but is not convincing evidence of same.

**We have added to the discussion, that lying awake quietly is a prerequisite of falling asleep (L332).**
